# The role of cytokines for clinical CAR-T cell manufacturing: systematic review and analysis of current evidence

**DOI:** 10.1038/s41551-026-01703-w

**Published:** 2026-06-29

**Authors:** Mar Guaza-Lasheras, Johanna Nimmerfroh, Dominic Schwarz, Tanja D. de Grujil, Jessica Hartmann, Christian Klein, Heinz Läubli, Michal Lotem, Joerg Mittelstaet, Christophe Paget, Sine Reker Hadrup, Sonja Schallenberg, Per Thor Straten, Sebastian Theurich, Dario Venetz, Alfred Zippelius, Laurence Zitvogel, Sebastian Kobold

**Affiliations:** 1 Department of Molecular Biotechnology and Gene Therapy, Paul-Ehrlich-Institut, 63225 Langen, Germany; 2 Division of Clinical Pharmacology, Department of Medicine IV, University Hospital, Ludwig Maximilian University (LMU) of Munich, Lindwurmstrasse 2a, 80337 Munich, Germany; 3 Department of Biomedicine, University of Basel; 4 Division of Oncology and Center for Cell Therapy and Immunomodulation (UZTI) University Hospital of Basel; 5 Ludwig-Maximilians-Universität München, Faculty for Chemistry and Pharmacy, 80337 Munich, Germany; 6 Roche Innovation Center Zürich, 8952 Schlieren, Switzerland; 7 Department of Medical Oncology, Cancer Center Amsterdam, Amsterdam University Medical Centers, De Boelelaan 1117, 1081HV Amsterdam, The Netherlands; 8 Sharet Institute of Oncology, Hadassah Hebrew University Medical Center, Jerusalem, Israel; 9 Miltenyi Biotec B.V. & Co. KG; 10 Institut National de la Santé et de la Recherche Médicale, Centre d’Etude des Pathologies Respiratoires (CEPR), UMR 1100, Université de Tours, Faculté de Médecine de Tours, Tours, France; 11 Department of Health Technology, Technical University of Denmark, Copenhagen, Denmark; 12 Center for Cancer Immune therapy (CCIT), Department of Oncology, Herlev University Hospital, 2730, Herlev, Denmark; 13 Ludwig-Maximilians-Universität München (LMU), University Hospital, Department of Medicine III, Munich, Germany and LMU Gene Center, Cancer and Immunometabolism Research Group, Munich, Germany; 14 Université Paris-Saclay, Faculté de Médecine, Le Kremlin-Bicêtre, France; Gustave Roussy, Villejuif, France; Institut National de la Santé et de la Recherche Médicale, UMR1015, Gustave Roussy, Villejuif, France; Center of clinical investigations BIOTHERIS, INSERM CIC1428, Gustave Roussy, Villejuif, France; 15 German Cancer Consortium (DKTK), Partner Site Munich, a partnership between the DKFZ Heidelberg and the University Hospital of the LMU, Germany; 16 Einheit für Klinische Pharmakologie (EKLiP), Helmholtz Zentrum München - German Research Center for Environmental Health Neuherberg, Germany; 17 Faculty of Life Sciences, Reutlingen University, Reutlingen, Germany

## Abstract

Chimeric antigen receptor T (CAR-T) cells represent a recent clinically validated modality in cancer therapy. An emerging frontier of this technology is the expansion of these curative treatments from hematological malignancies to solid cancers and autoimmune diseases. To date 1627 CAR-T clinical trials are registered in clinicaltrials.gov. Rapid and broad scale industrial implementation requires adequate safety considerations, logistics, regulatory implementation and financial viability. A major bottleneck of CAR-T therapy is the *ex vivo* production process, where cytokines are an essential and product determining constituent. Here, we review how the use of cytokines in CAR-T manufacturing has historically evolved, examining their roles in cell expansion, activation and impact on the overall therapeutic efficiency both pre-clinically and clinically. We extracted extended metadata from 296 available clinical reports at data cut off, to elucidate the global role of cytokine utilization throughout the production process. Our analysis focuses on surfacing relationships that could inform on the effectiveness of different cytokine compositions and prevent redundant clinical trials offering limited advancements. The findings from this review and meta-data analysis are intended to provide insights that could lead to the standardization of cytokine-use in CAR-T cell manufacturing, ultimately improving process efficiency and reducing associated costs.

## Introduction

Immunotherapies have revolutionized cancer treatment by harnessing the power of the patient’s immune system to target and eliminate tumor cells. T cell engaging bispecific antibodies (TCB) and chimeric antigen receptor modified T (CAR-T) cells have emerged as two clinically efficacious modalities based on unparalleled clinical response rates and long term outcomes^1,2^. TCBs such as blinatumomab targeting the B cell-associated surface antigen CD19 were the first to clinically demonstrate the potential of T cell redirection. Later, CD19-directed second-generation CAR-T cell products (CD19-CAR T cells) showed similar efficacy and long-term curative effects in patients suffering from hematological malignancies^3^. Awakened by these encouraging outcomes, >200 cell therapy trials are starting each year to tackle hematological malignancies and solid tumors^4^, illustrating the extreme dynamics of this vibrant field.

A major goal remains to bring the full potential of cellular therapies to patients affected by solid tumors and beyond. Along these lines, researchers strive to improve their cell products by optimizing manufacturing and cell engineering processes. A key determinant for the success of CAR-T manufacturing and therapeutic efficacy is the use of specific cytokines during CAR-T cell generation. Cytokines are proteins with crucial functions related to T cell proliferation, differentiation, survival and homeostasis. Cytokines present an essential component of CAR-T cell manufacturing process and allow multifold expansion of T cells necessary to achieve a meaningful clinical dose and response after adoptive transfer. However, cytokine usage is not standardized and there is limited knowledge about their use at the global scale, including the potential influence of differences and disparities on clinical outcome.

In this review, we aim to provide an overview of historical and current clinical developments concerning the use of cytokines during CAR-T cell therapy. We further performed a data-driven analysis and interpretation of the publicly available subset of clinical-stage CAR-T cell trials with a focus on their detailed manufacturing parameters. Therefore, we conducted an extensive PubMed search, to collect 296 trial reports, which span 18 % of the global 1627 CAR-T cell trials. Manufacturing details including starting material, gene engineering vehicle and cytokine use, but also response rates and toxicity scores were extracted from primary- and secondary sources. Next, the data was supplemented with trial details from clinicaltrials.gov and unbiased meta-analysis was conducted. We display historic and global trends as well as correlations of recorded parameters. This dataset uncovers shared patterns within an increasingly diverse manufacturing landscape, which is often shrouded in trade secrecy.

To our knowledge, this is the largest collection of CAR-T cell manufacturing parameters. This work is intended to inform researchers of all stages and serve as an extendable atlas that will provide a common ground to reduce complexity and costs of CAR-T manufacturing processes to accelerate patient access.

### History of CAR-T cells

Tumor infiltrating lymphocytes (TIL) were the first therapeutic lymphocytes to be deployed clinically as naturally occurring anti-cancer effector cells^5^. A few years later, first-generation CAR constructs were proposed, fusing a scFv fragment to the CD3ε domain to redirect T cells to recognize antigens independently of the major histocompatibility complex (MHC)^6^. Despite promising data generated in mouse models, these cells failed to persist and to control tumors in patients as reported in first clinical studies on ovarian cancer and metastatic renal carcinoma in the early 2000s^7–9^.

Physiologically, T cells require two signals for activation: the first one resulting from engagement of the T cell receptor (TCR) with its peptide-loaded major histocompatibility complex and the second one induced by costimulatory receptors, such as CD28^10,11^. In 1998, CD28 was first introduced as a costimulatory domain into CAR-design, resulting in increased persistence in contrast to first generation constructs^12,13^. With a similar rationale, 4-1BB was incorporated as an alternative costimulatory signal into second-generation CAR-T cell constructs, showing improved persistence and anti-tumor efficacy in mice as compared to constructs bearing CD28^14^.

The first clinical trial using CAR-T cells was published in 2011, showcasing potent anti-tumor effects of CD19 CAR-T cells on adult patients with chronic lymphocytic leukemia (CLL)^15,16^. In 2012, the first ever pediatric ALL-patient was treated with CD19 CAR-T cells and achieved complete remission with no signs of disease to this day^17^. This patient became the face of CAR-T cell promise and is widely known even by the general public. This success was reinforced shortly afterwards by others^18^ and led to the development and approval of Kymriah, the first approved CD19-targeted CAR-T product for the treatment of pediatric and young adult ALL. There are currently seven approved CAR-T cell products targeting either CD19 or B cell maturation antigen (BCMA) to treat multiple patient indications, including B cell acute lymphoblastic leukemia (ALL), B cell non-Hodgkin lymphoma (NHL), follicular lymphoma, mantle cell lymphoma (MCL) and multiple myeloma^19^.

In solid tumors progress has been more challenging, but recently successful Phase 1 clinical trials were reported targeting e.g. CLDN18.2, CLDN6, GD2, PSCA or GPC3^20–25^. CLDN18.2-CAR-T cells were tested in a phase 1 trial resulting in 44,8% overall response after 6-months, showing promising efficacy with an acceptable safety profile in patients with heavily pretreated, CLDN18.2-positive digestive system cancers, particularly in those with gastric cancers^20^.

### CAR-T cell manufacturing

The clinical success of CAR-T cells has led to the initiation of clinical trials, with contributions from both pharmaceutical companies and academic centers. All currently commercialized products are produced in centralized manufacturing sites. In contrast, many of the early-stage clinical trial products initiated by academic centers are produced in decentralized manufacturing sites at the point of care (POC)^26^. While decentralized manufacturing offers potential advantages, such as leveraging hospital infrastructure, obviating cryopreservation, and reducing vein-to-vein time, it also introduces significant challenges. These include limited standardization, variability in operator expertise, and restricted automation, which can result in products of inconsistent quality and potentially impact patient outcomes^27,28^.

All manufacturing steps need to follow strict good manufacturing practice (GMP) guidelines enforced by the respective local regulation regulatory authorities. These guidelines regulate only over the actual manufacturing processes but also over the quality grade of ancillary components such as cytokines, activating beads/antibodies and culture media^29^. Compliance with GMP guidelines is critical for ensuring safety and consistency of CAR-T cell products, minimizing the risk of contamination, adverse reactions, or variability in therapeutic outcomes. The regulatory frameworks for manufacturing and market authorization differ depending on the regulatory region. The FDA (Food and Drug Administration) is the regulatory agency in the US, EMA in Europe (European Medicines Agency) and NMPA (National Medical Products Administration) in China. The FDA classifies CAR-T cells under cell therapies and CAR-T cells in Europe are categorized under Advanced Therapy Medicinal Products (AMTP)^29^. Both EMA and FDA have their specific regulatory recommendations for the development of CAR-T cells, but main regulatory milestones are comparable^30^.

For manufacturing of CAR-T cells according to GMP standards, a variety of materials are commercially available. Individual components were recently described and will be discussed in greater detail in the data analysis section of this review^31,32^.

**Figure 1:**
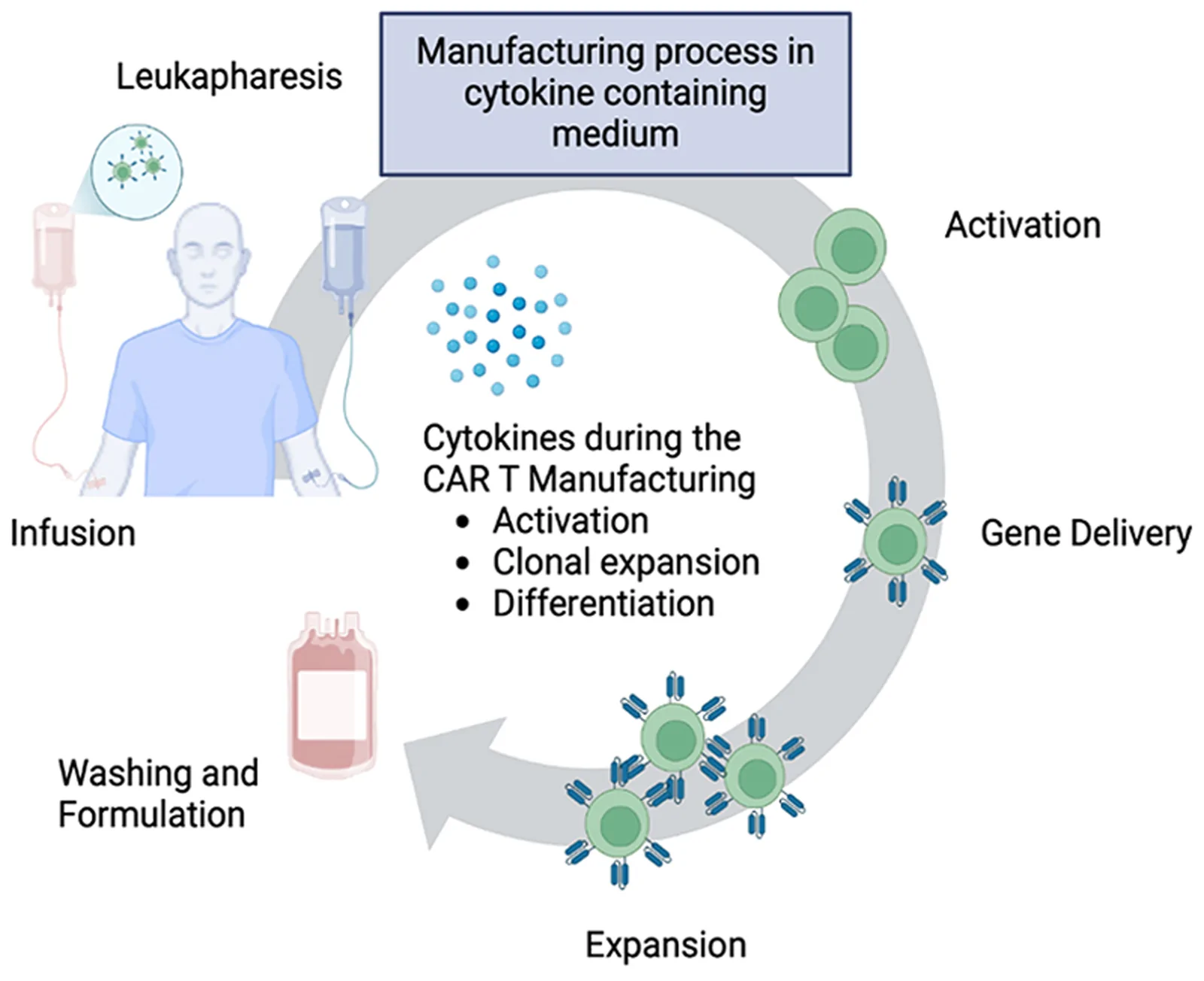
Schematic of the CAR T manufacturing process For CAR-T cell manufacturing, T cells are commonly obtained via leukapheresis. Collected T cells are cultured, activated and transduced with the CAR transgene. CAR-T cells are further expanded in culture, until final formulation and administration into the patient. Conventional CAR-T cell manufacturing involves 1–2 weeks of ex vivo manipulation and expansion, with all steps conducted in cytokine-containing media.

CAR-T cell therapy consist of a multi-step process by which a patient’s T cells are isolated, genetically modified, expanded, and re-infused into the patient, resulting in a highly personalized cancer treatment^32^ (Figure 1). The individualized steps are detailed bellow.

Initially, T cells are collected through a procedure called leukapheresis, either from the patient her- or himself in an autologous setting or from a healthy individual for allogeneic CAR-T manufacturing^27^. Next, T cells are cultivated and activated to expand them to sufficient numbers. Activation of T cells is required to permit efficient viral gene delivery^30^. Combination of anti-CD3 and anti-CD28 antibody-based stimulation induces efficient clonal expansion of CD4+ and CD8+ T cells and serves as a core element of CAR-T cell manufacturing^33^. Other earlier approaches relied on expansion of T cells in a cell-dependent approach with feeder cells^34^ for T cell stimulation. Antigen-presenting cells (APCs), such as dendritic cells (DC) or irradiated PBMC were used as an endogenous activator of the T cells. However, propagation of some antigen-presenting cells proved to be inconsistent and extremely difficult to generate in adequate numbers across patients^32^. Second, some cell types can be immunosuppressive *in vitro* and limit the desired functionality of T lymphocytes. Artificial antigen-presenting cells, such as transduced and irradiated K562 APCs have been employed, but their generation in GMP grade remains complex (NCT02807883)^32,35^. Along these lines, antibody-based stimulation has largely outpaced feeder cells in CAR-T cell generation.

Stable CAR expression can be achieved via viral and non-viral T cell engineering methods. Viral gene delivery utilizes γ-retroviral or lentiviral vectors, which are produced in cell systems optimized for viral yields. This results in infectious but replication-deficient viruses with varying tropisms. However, viral methods are limited by high costs associated with manufacturing these viruses under GMP conditions, limited cargo capacity and risks of immunogenicity^36^. Non-viral approaches include chemical or electroporation delivery methods, such as transposon/transposase systems, and CRISPR-Cas9 engineered CAR-T cells, which have been employed in more recent trials. Notably, these systems present lower insertional risks compared to viral vectors (REF). Transposon-based systems such as Sleeping Beauty or Piggy Bac make use of transposons and transposases that can be vectorized as mRNA and DNA. Similarly, endonuclease-based systems, such as CRISPR-Cas9, can also be vectorized. The relatively simpler production process of these reagents in GMP quality may results in overall lower costs and accessibility. In contrast, messenger RNA transfer comes with short-time gene expression, which is generally not beneficial in a cancer context and is therefore less widely used^37^.

CAR-T cells are further expanded, washed, and ultimately formulated for patient administration. In recent years, closed systems have been developed to decrease production complexity of an otherwise manual process and to mitigate risks of contamination and errors caused by human manipulation^38^. Development of closed-system manufacturing makes CAR-T cell production more accessible to academic centers, by reducing manual labor and the need for high-level GMP environments. GMP systems are classified based on the grade of cleanliness of the production environment. In most countries, systems can be run in a C or D level environment, while the manual process requires A or B^34^. These systems typically include automated bioreactors, closed centrifugation devices, and tubing systems designed to maintain a sterile, controlled environment throughout the manufacturing process. By minimizing manual interventions, closed systems enhance process reliability and compliance with GMP standards.

Throughout the entire CAR-T cell production process, T cells are *ex vivo* cultivated in cytokine-containing media to ensure their expansion, differentiation and survival. Despite variations across the manufacturing protocols evaluated in this review, this practice is a consistent feature across all protocols during each production step until collection of the final product. This underscores the pivotal role of cytokines to ensure both the quality and therapeutic efficacy of the CAR-T cell product.

### Developments of cytokine-use during CAR-T cell development

Rapid and efficient expansion are integral determinants of CAR-T manufacturing success. Along with cell purity, transduction efficiency, sterility and therapeutic cell doses, these factors ensure that patients can clinically benefit from CAR-T cell therapy. Several strategies have been deployed in the past for optimization of expansion protocols and we give an overview of key advancements in the following paragraph.

**Figure 2:**
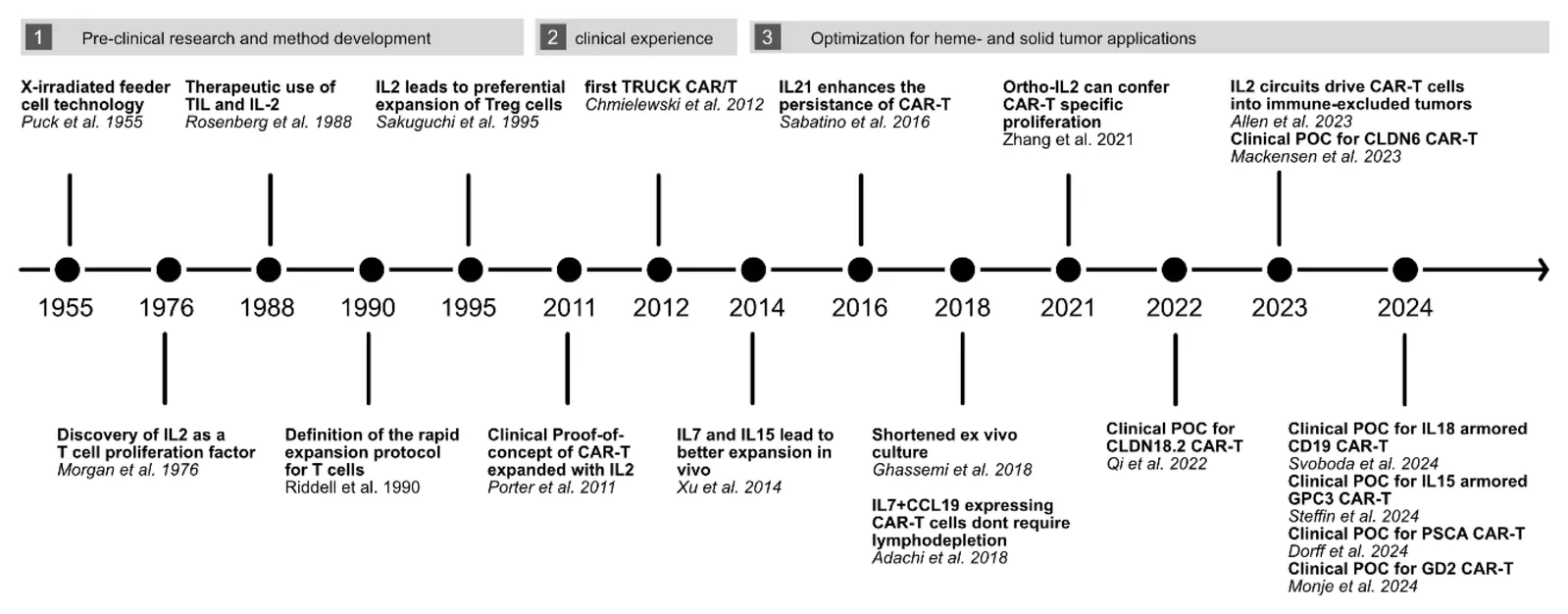
Graphical overview of key developments in CAR-T manufacturing and cytokine use over time

It is hard to believe that just 70 years ago, culturing any non-cancerous cell types was a major challenge, if not impossible (Figure 2). Before the discovery of IL-2, the use of conditioned supernatants or irradiated feeder cells were the main available options to culture lymphocytes. Back then, it was only hypothesized that such cells secrete unknown growth factors that are required for proliferation beyond essential nutrients. In 1955, irradiated feeder cells were discovered to provoke proliferation of other cells^39^. Later, Phytohemagglutinin (PHA) stimulated human lymphocytes provided a conditioned medium permitting selective growth of T cells. Such media was postulated to contain a T cell growth factor, which was subsequently identified as IL-2, a factor imperative for T cell proliferation^40,41^. Shortly thereafter, the first cancer patient could be cured with high-dose IL-2 as a form of immunotherapy. Consecutively, IL-2 has been routinely applied in the context of TIL therapy and is also approved for the treatment of metastatic renal cell carcinoma and metastatic melanoma^42–44^.

The effect of IL-2 is limited amongst others, by regulatory T cells (Treg). Tregs are a suppressive cell population that expresses high levels of IL-2-receptor alpha (CD25) to bind IL-2 with high affinity^45^. Preferential IL-2 binding scavenges this key cytokines from inflammatory lymphocytes such as T cells, while expanding Treg, which can exert further inhibitory actions by secreted or membrane bound factors (reviewed in *Chinen* *et* *al*.^46^)^45,47,48^. High IL-2 concentrations decrease early memory T cell frequency and Treg formation while boosting effector T cells^49^. Prolonged IL-2 exposure in culture promotes terminal T cell differentiation and exhaustion, both of which negatively correlate with T cell therapeutic functionalities^50^. This outlines that there is a strong preclinical rationale to optimize CAR-T cell manufacturing as well as to reduce *ex vivo* expansion time for selection of more naïve and multifunctional T cells^51^.

**Figure 3:**
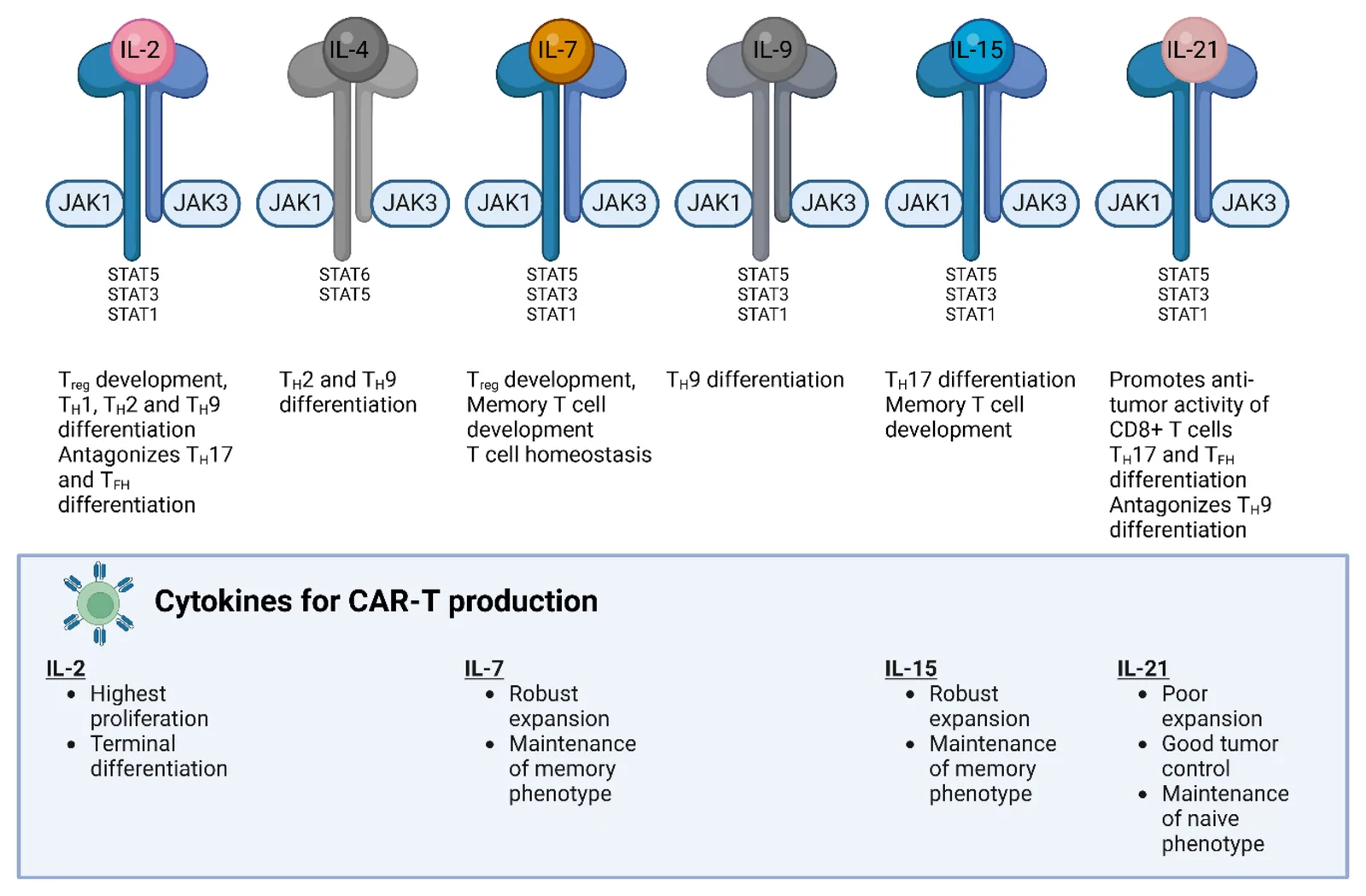
Common γ-chain cytokines and their use in CAR-T cell manufacturing Illustration of the interleukin (IL) receptor signaling pathways relevant to CAR-T cell production, focusing on gamma chain (γc)-dependent cytokines. Each cytokine (IL-2, IL-4, IL-7, IL-9, IL-15, and IL-21) binds its respective receptor, upon binding the receptor dimerizes with the common γ chain (γc, CD132). Ensuing signaling via JAK/STAT pathway involves trans-activation of JAK kinases and tyrosine phosphorylation. IL-2 can bind to the IL-2Rα (CD25) and IL-2Rβ (CD122; IL-4 to the IL-4Rα, IL-7 to the IL-7Rα (CD127); IL-9 to the IL-9R (CD129); IL-15 to the IL-15Rα and IL-2Rβ (CD122, shared with IL-2 receptor); IL-21 to the IL-21R. Downstream signaling leads to distinct T cell phenotypes actionable for CAR-T cell production. While IL-4 and IL-9 are included for context, they are not commonly utilized in CAR-T cell manufacturing.^55–57^

Proliferation is a common downstream effect shared by all common γ-chain family cytokines when applied to *ex vivo* culture of T cells (Figure 3). Beyond IL-2, cytokines like IL-7, IL-15 and IL-21 may additionally induce alternate differentiation pathways that could potentially yield better CAR-T cells for some applications^52^. Members of the common γ-chain cytokine family bind to the common γ-receptor through interactions with specific epitopes, which vary in affinity and contribute to the receptor’s functional diversity^53^. This intriguing finding offers insights into the remarkable overlap and pleiotropism of the common γ-chain cytokines (IL-2, IL-4, IL-7, IL-9, IL-15 and IL-21). Pleiotropism is defined as the ability of a cytokine to exert a wide range of different effects on different cell types and states^54^. Depending on their differentiation state, T cells can differentially modulate the level of receptor surface expression and thus regulate their receptiveness to a respective cytokine. This functional diversity is only outlining a fraction of the complex network of regulatory mechanisms that may be subject to optimization for CAR-T cell manufacturing workflows.

In detail, IL-7 acts as a thymic survival signal and homeostatic regulator of naive and memory T cells^58^, IL-4 can induce Th2 or Th9 differentiation of CD4+- T cells, IL-9 confers proliferation to Th17 cells, and IL-2, IL-15 and IL-21 regulate proliferative effects on CD4+- and CD8+- T cells^59,60^. IL-15 was shown to specifically reverse anergy^61^ and increase resistance to inhibitory effects of Tregs^62^ in an antigen independent manner.

Translational studies revealed that patients with B cell malignancies, responding to CAR-T cell therapy were treated with a product of high T-memory stem-cell-like cell percentage (CD8+CD45RA+CCR7+)^63^. Consequently, to overcome previously mentioned limitations of IL-2 use, IL-7, IL-15 and IL-21 were leveraged in preclinical CAR-T cell cultures to enrich this subset and confer enhanced anti-tumor activity. A limited set of preclinical comparative studies present a nice overview over the complex combinatorial potential of cytokines during manufacturing: IL-2, IL-7 and IL-15 confer the greatest proliferative potential *in vitro*, compared to IL-21, however, only IL-7, IL-21, but not IL-2 or IL-15 cultured T cells show comparable T cell activity in mice^64^. IL-21 emerged as an appealing candidate that may also offer opposing differentiation programs to IL-2^65^. IL-21 conditioned T cells experience greater secondary expansion after antigen rechallenge than IL-2 or IL-15 primed cells^66^. IL-21 may also confer ameliorated CAR-T cell persistence *in vivo*, a phenomenon shared with IL-7 and correlating with better outcome in murine experiments^67^. A direct clinical comparison of CD19 CAR-T cells either manufactured with IL-2 versus IL-7/IL-15 has so far only been performed in a handful of trials and results have yet to be reported (NCT00586391 and NCT00709033)^63^. One very recent study indicates an opposing perspective on the discrimination of IL-2: the investigators switched mid-trial between IL-7/IL-15 and IL-2 conditioned media and observed a relevant increase in CAR-T cell long-term persistence in patients when manufactured with IL-2^68^.

Other, non-common γ-chain family cytokines such as IL-12 and IL-18 have already received significant interest. Both cytokines are not considered to confer antigen-independent proliferation and have distinct signaling pathways. IL-12 does not induce proliferation directly but IL-12 conditioning during *ex vivo* culture can lead to increased Th1 differentiation and CD28+CD62L+ T cells^69^. Both T cell phenotypes are associated with enhanced *in vivo* functionality and outcome^70,71^. In addition, T cells conditioned with combinations of IL-12 and other cytokines (e.g., IL-12 and IL-7 or IL-12 and IL-21) may show better engraftment when compared to IL-2^69^. In contrast, IL-18 stimulation can have proliferative effects and does so via secondary mechanisms^72,73^. IL-18 influences production of IFN*γ* thus conferring preferential differentiation into a Th1 subtype and increased autocrine IL-2 production^72^. Although it remains speculative whether all these findings will have a similar outcome in combination with CAR and across indications, early studies suggest a similar pattern.

In summary, although the use of cytokines in manufacturing of CAR-T cells has shown considerable promise in preclinical studies, seamless translation of new findings and advances into clinical settings - although compelling - is a subject of active research.

### Future avenues of CAR-T manufacturing

Cytokines may also hold great value, when engineered directly into- or specifically for CAR-T cells. In principle, the use of cytokine receptor signaling moieties can be distinguished from induced or constitutive cytokine secretion. Armored CAR-T cells commonly refer to constitutive secretion, whereas TRUCKs (“T cells redirected for universal cytokine-mediated killing”) refer to CAR-T cells secreting immunomodulatory cytokines in a tumor-activated manner.

Several concepts exploiting this basic principle are in an advanced stage of development. For example, CAR-T cells engineered to secrete IL-7 and CCL19 are currently in phase 2 clinical trials in China (#NCT03929107). Preclinically, these CAR-T cells were shown to act as a spark to ignite the fire: By engaging with dendritic cells (DC), deep tumor tissue infiltration of T cells and DCs resulted in rechallenge-resistant complete cures in mouse models^72^. IL-7 is presumably acting in an autocrine fashion, thus systemic IL-7 concentration and associated leukemic potential is not increased. Additionally, epitope spreading contributed to engage an endogenous TCR-mediated T cell response, allowing rejection of antigen-negative tumor cells. In fact, the transient in vivo depletion of circulating T cells after IL-7 administration has been ascribed to the capacity of IL-7 to upregulate the α4β7 receptor of the mucosal addressin MAdCAM-1 involved in intestinal homing and memory-like conversion of naïve T cells observed in the IL-7–driven immunological reconstitution post-lymphopenia^74^. Homing of CAR-T to extratumoral compartments may deserve further attention.

Another noteworthy clinical example of cytokine-armoring are GPC3-targeted CAR-T cells that secrete IL-15 (NCT05103631 and NCT04377932)^25^. The authors suggest that IL15 preferentially improves cell expansion in an autocrine manner, because IL-15 serum concentrations were not higher compared to systemic administration^75^. Notably, anti-tumor effects were more frequent (disease control rate of 66 %), despite lower cell concentrations compared to earlier GPC3 CAR-T cell trials^76^. Although CRS-related side effects were more common using this novel product they remained manageable by IL-6/IL-1 blockade^77,78^ or use of a safety switch^79^.

Other studies focused on cytokines mostly acting on the TME that can be conditionally expressed upon T cell activation: such as IL-12 and IL-18^80–82^. IL-12-TRUCKs demonstrated impressive preclinical efficacy and could offer the possibility to skip prior patient conditioning. However, clinical trials using such CAR-T cells were terminated for lack of efficacy and unexpected toxicity in patients (#NCT01236573 and #NCT01457131). Similarly, engineered TILs with an inducible IL-12 also lead to severe toxicity in melanoma patients, despite up to 100-fold lower TIL dose than conventional applications^83^.

IL-18-armored CAR-T cells have received particular attention in recent years. IL-18 may be a cytokine with compelling characteristics for CAR-T cell therapy: It is a proinflammatory cytokine that synergizes with IL-12 to produce Th1 responses and increase cytotoxicity^84^. This effect is driven by production of IFNy, an important factor required for the treatment of solid tumors^85^. Unlike IL-12 however, the combined safety profile of CAR and IL-18 seems to be more acceptable^86^. This concept is evaluated pre-clinically and clinically, targeting CD19 and GD2, respectively (#NCT06287528 and EU CT 2022-501725-21-00)^86,87^.

These clinical results indicate the need of controlling these payloads and their release and may open the door to more sophisticated logic-gated designs. A precise response to tumor-intrinsic or synthetic cues may improve spatial precision of CAR-T cell activity and thus enable better safety profiles.

In preclinical development, a human orthogonal IL-2 and IL-2Rb system allows targeting of IL-2 specifically to engineered CAR-T cells while having no effect on other lymphocytes^88,89^. This technology overcomes the need for lymphodepleting chemotherapy, which is currently a necessity for expansion and engraftment in a recipient organism. Another preclinical orthogonal technology is a synthetic IL-2 exhaustion circuit^90^. Here, a synthetic Notch receptor induces secretion of IL-2. This technology enables T cells to infiltrate immune excluded tumors and thus achieve strong curative potential and can potentially circumvent tumor immunosuppression in mouse models.

Another potential strategy to maintain CAR-T cell recirculation and functions leverages the gut barrier and microbiome fitness and diversity. Several studies indicated that the microbiota taxonomic composition can influence CAR-T cell persistence and side effects^91,92^. The mechanisms involved in the cross-talk between the intestinal content and the peripheral systemic tonus include distinct metabolites (short chain fatty acids^93^, indoles, inosine^94^) involved in T cell fitness or autophagy and trafficking of CAR-T to specific compartments (such as bone marrow). The role of cytokine signaling in CAR-T cell metabolic rewiring is under study.

The above-mentioned negative aspects of rapid *ex vivo* CAR-T expansion have provoked research into alternative avenues to improve manufacturing. For instance, multiple studies have already shown that shorter *ex vivo* expansion may yield a superior cell product^95–97^. Reduction of the expansion process from 9 to 3 days leads to a product with enhanced proliferative capacity and improved persistence in preclinical models^98^. Such a rapid manufacturing process may be further reduced to just one day using non-activated T cells and is functional in murine models and patient samples^96^. This important development has a two-fold rationale: A better cell product may be achieved by transferring T cell expansion phase into patients, rather than in culture, rendering the use of cytokine cocktail optimization less critical. Additionally, the impact of shortening production processes and its financial implications may facilitate widespread clinical application of this technology. Although promising, some risks of cytokine release syndrome (CRS) have been reported after short-term manufactured CAR-T cell administration^99^. Whether such methodology can replace existing CAR-T manufacturing processes will need to be demonstrated in clinical trials.

Another alternative to shortening *ex vivo* production times is the generation of CAR-T cells directly *in vivo*. Firstly, patients do not have to undergo lymphodepletion treatment pre CAR-T treatment, since targeted cells are the patient’s own lymphocytes. Secondly, patient’s T cells do not need to be extracted, handled and expanded *ex vivo*, which can result in less T cell exhaustion and thus better performance^95,100,101^. A major obstacle for this avenue is to selectively target only the patient’s T cells and not any malignant cells, as this has already been reported to potentially result in CAR+ therapy resistant second primary malignancies and even patient death^102^. In order to surpass that hurdle, researchers have turned to cell-targeted viral vectors as vehicles for introducing the CAR cassette specifically, to targeted lentiviral vectors or lipid nanoparticles (LNPs) although adeno-associated-viral vectors (AAVs) are also gaining momentum. Concomitant to our literature search, a single company has managed to get approval for testing *in vivo* CAR-T cells in a clinical setting. In fall 2024, Interius BioTherapeutics launched and dosed its first patient with CAR T cells targeting CD20 B cell malignancies generated *in vivo* with CD7-targeted lentiviral vectors (NCT06539338). As with short-term *ex vivo* generated, *in vivo* generation of CAR-T cells has also been linked to risk of CRS^103^.

It is becoming increasingly clear that next-generation CAR-T cell products will need cytokine signals to complete the triad of T cell activation signals and thus confer complete T cell activation. As manufacturing processes become shorter, including the generation of CAR T cells from non-activated T cells, CAR-T expansion process may eventually take place in patients^97^. This necessitates that CAR-T cells engraft in a host and receive a strong but fully controllable proliferation signal and finally form a memory compartment to prevent relapse.

## Methods

We performed an extensive PubMed and ClinicalTrials.gov research to map the landscape of clinical CAR-T cell trials. The data cut-off was set to 15.10.2024.

Raw clinical trial information was collected, such as NCT No., date, status and phase, from CAR-T cell interventional studies from ClinicalTrials.gov using the MESH term: *“CAR-T” OR “chimeric antigen receptor” OR “chimeric T-cell receptor” OR “Receptors, Chimeric Antigen”*. This resulted in a total number of 1891 studies, of which 264 were falsely annotated and manually excluded. Only studies where the intervention is a CAR-modified cell were included and due to their scarcity, CAR-NK, CAR-M, CAR-DC and other non-T cell products were not excluded.

We systematically screened the PubMed database to identify clinical trials involving CAR-T cell therapies. CAR T related MESH terms: “CAR-T” OR “*chimeric antigen receptor” OR “chimeric T-cell receptor” OR “Receptors, Chimeric Antigen”*, were used and results were filtered for “*Clinical trial”*. In this process 432 publications surfaced. We manually screened publications and filtered for studies with the following inclusion criteria: 1. peer reviewed clinical studies; 2. published until October 2024; 3. available in the English language. We excluded 1. duplicated trials, 2. preclinical papers, 3. studies without a registered trial number and 4. publications falsely indexed as CAR-T related. 296 unique publications were selected for meta-data extraction based on these criteria. Manufacturing details, if only referenced in the primary publication, were extracted from secondary literature, linked protocols and supplementary information, as well. We additionally assessed clinical trial registration platforms (clinicaltrials.gov, chictr.org.cn) of the corresponding clinical trials for study information. We collected in tabularized format 1. baseline characteristics of included studies (first author, last author, year of publication, date of clinical trial registration, country of trial registration, center of clinical trial, registration number, status of trial, trial phase); 2. details on CAR-T cells (target, format, intracellular domains); 3. clinical information (type of disease, clinical response, toxicities); 4.if publicly available, we collected manufacturing details (media, starting material, cytokines, activation methods, culture times, promoter, culture vessel)

Collected data contain information about target, structure, disease indication, geographical and chronological parameters. In case of fluctuating cytokine concentrations or adaptations in the manufacturing protocol, we opted to consider the highest concentration mentioned for data analysis.

## Results

**Figure 4:**
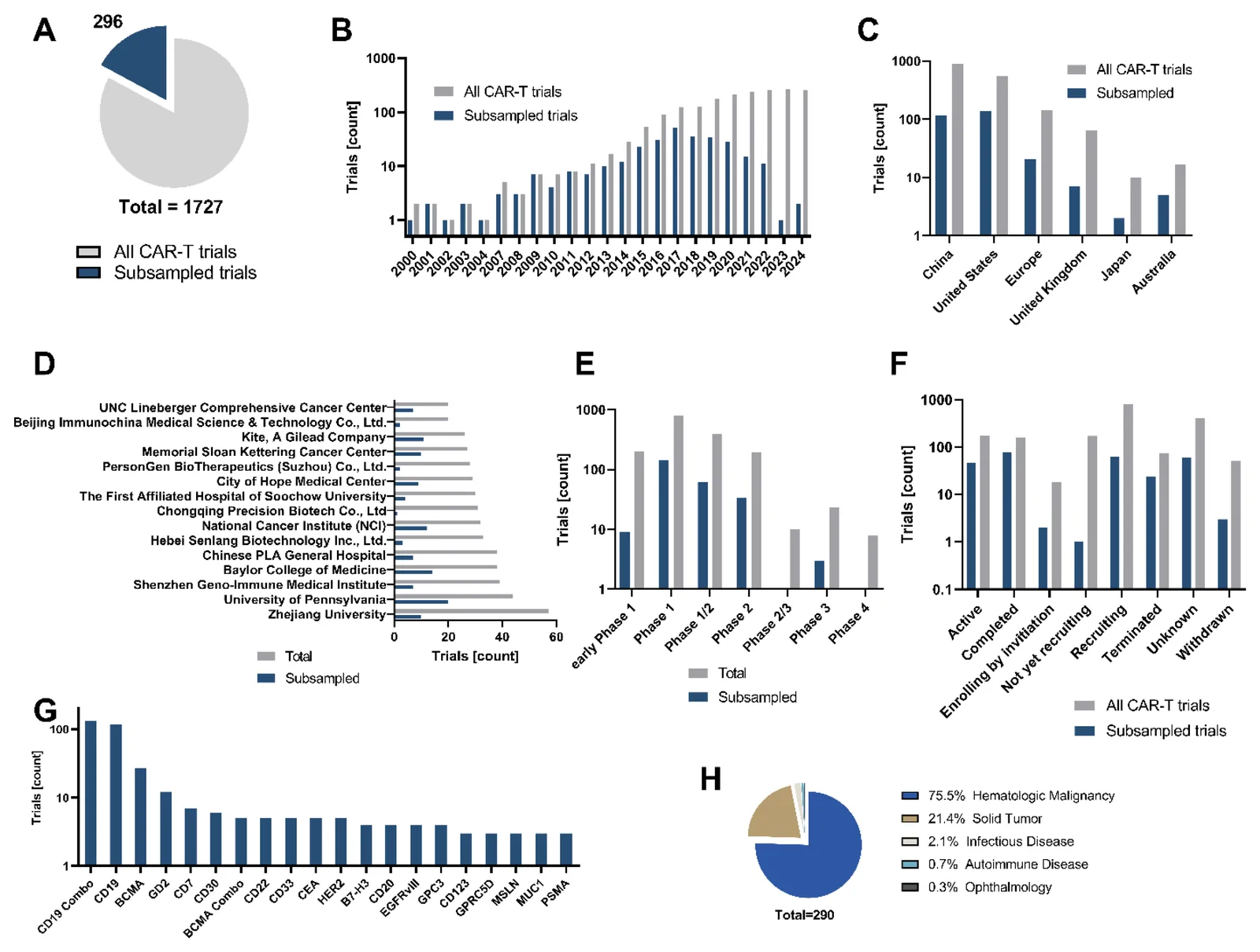
Comparison of all cell therapy clinical trials to a subsampled cohort of 296 trials A) Pie chart showing the number of subsampled trials compared to all registered CAR-T. The amount of trials B) per year, C) per regulatory region and D) per study sponsor (in order of most studies done across all CAR-T trials), E) per clinical phase and F) per trials status is portrayed to illustrate the representativeness of the subsampled cohort. G) Overview of subsampled CAR-T cell trials and the cellular antigens targeted in order of count. There are >30 additional studies targeting unique antigens. H) Pie chart showing the percentage of disease area targeted in the subsampled dataset.

As of 15.10.2024, a total of 1727 CAR-T cell therapy clinical trials were registered on clinicaltrials.gov. We subsampled all PubMed articles that report on clinical CAR-T cell therapy results to provide an overview on manufacturing details and study information published in peer-reviewed journals, as further described in the methods sections of this review. Unlike information commercially available and meta information quickly accessible from general clinical trial information, this dataset includes tabularized additional information about CAR format, costimulatory domain(s), response rate and toxicity. We complemented the dataset with information about manufacturing of associated CAR-T products, including but not limited to: starting material, T cell activation method, culture medium, serum type and concentration, the type of gene delivery tool used and cytokines as well as their concentrations. On average, this information is available in less than 50 % of trials. When it was publicly available, information about culture times, culturing plates and promoters was also recorded. Notably, this information is only available in an even more limited number of study-associated publications, given that it is treated as a business secret by many, sometimes even among academic groups. After an initial screening, 296 interventional trials fulfilled the criteria for downstream analysis and were selected for our extraction of metadata and analysis (Figure 4a)

First, we wanted to find out if the subsampled dataset has comparable proportional distribution and could thus be used as a representative cohort to inform on all global CAR-T cell therapy trials. To this end, we used primary trial characteristics such as the initiation date of the trial, the country, the sponsor and the trial phase, which are available for all CAR-T cell therapy trials and the subsampled cohort. The subsampled dataset encompasses clinical trials conducted between 2000 and 2024 (Figure 4b) including studies performed throughout the entire period of CAR-T trial registration. Data coverage is higher in the early years of CAR-T clinical trials due to the time gap between trial registration and final reporting. The subsampled trials have a certain temporal bias, which is inherent to reporting and academic publishing.

Geographic origin of the clinical trials was determined based on the location of the study center and investigator (Figure 4c,d) and further clustered into regulatory regions. Overall, most trials were run in China (118/296 studies, 39.9 % of subsampled trials vs. 912/1727, 52.8 % of total studies) and the United States (137/296, 46.3% of subsampled studies vs. 550/1727, 31.8 % of total trials), followed by Europe (21/296, 7.1 % vs. 142/1727, 8.2%), United Kingdom (7/296, 2.3% vs. 64/1727, 3.7 %), Australia (5/296, 1.7% vs. 17/1727 1.0%), Japan (2/296, 0.7 % vs. 10/1727 0.6 %), and others (<<1 %). Notably, the number of trials performed in the United Kingdom (7 samples vs 64) is over proportional compared to those run by the rest of Europe combined (21 subsamples vs 142 total). The subsampled trials cover studies of all mentioned regulatory regions reflecting 15-25 % of total trials and are thereby a representative geographical cohort. We must concede that results from pharma companies and Chinese academic research centers may be underrepresented. The ratio between registered trials and reported trials is lower compared to other countries.

The subsampled dataset reports trials conducted from early phase I trials up to phase III (Figure 4e). Late-stage clinical trials are either exclusively big pharma companies sponsored and/or run in China, or manufacturing details are treated as business secrets. The time shift in reporting has additional impact on bias towards early-stage clinical trials. Among the subsampled cohort, 79/296 (26.7 %) of the sampled trials were completed (vs.157/1727, 9.1 % total), 47/296 (15.8 %) were active (vs. 176/1727 10.1 %) and 66/296 (22.2 %) were actively recruiting (vs 813/1727, 47.1 %). Overall, 60/296 trials are marked with unknown status (20.2 % vs. 408/1727, 23.6 %) (Figure 4f). Due to the nature of the data collected, the subsampled dataset covers all mentioned statuses but underrepresenting late-stage clinical trials.

Within the analyzed cohort, all 296 studies disclose their target (Figure 4g). 61 different targets were identified as follows: CD19 (116/296 trials, 39.2 %), followed by BCMA (27/296 trials, %) trials) CD19 dual-CARs combined with either CD20, CD22, BCMA, CD123, CD7 (28/296 trials, 9.5 %) and GD2 (12/296 trials, 4 %). 283/290 (97.6 %) of subsampled trials investigate CAR-T cells in the context of malignant diseases (Figure 4h). 216/290 (74.5 %) of trials used CAR-T cells to treat hematological malignancies, followed by 62/290 (21.4 %) (targeting solid tumors. A small subset of trials (3 %) investigate CAR-T cell therapy for infectious (6/290, 2.1 %) or autoimmune (2/290, 0.7 %) diseases.

In summary, a very similar proportional distribution between the subsampled accessible study cohort and all global active or planned CAR-T cell therapy clinical trials was observed. We reason that the present included data set will thus be able to draw conclusions of use to the full cohort and community.

Initially, we performed a descriptive analysis of the accessible study cohort. As we have previously established some confidence of correlation to all clinical trials, we assume that this dataset of extended manufacturing parameters can inform on global trends as well.

**Figure 5:**
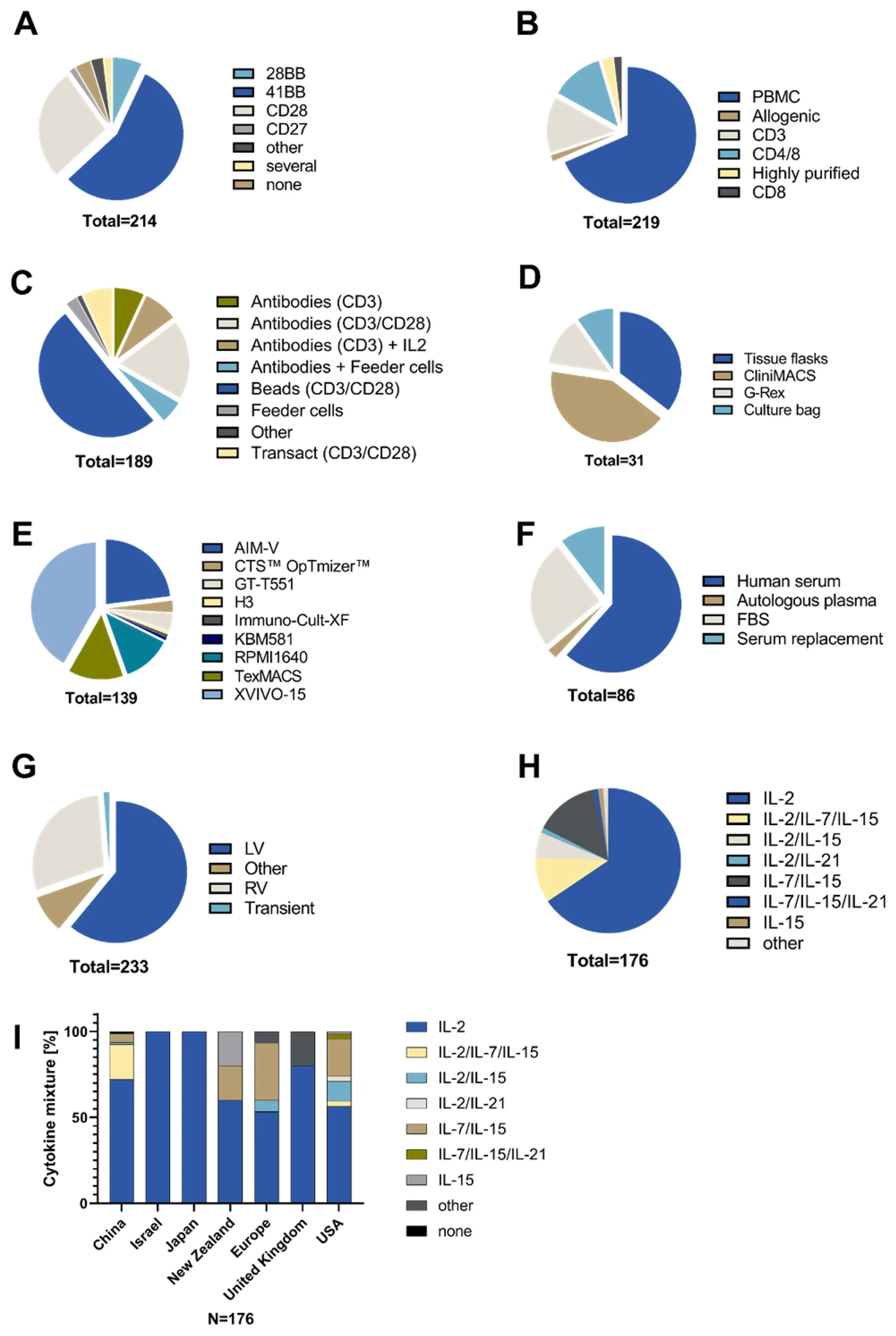
In-depth analysis of the cell therapy manufacturing processes in a subsampled trial cohort Plots showing A) costimulatory domains used in the subsampled dataset. B) Starting material used for CAR-T manufacturing. C) T cell activation cocktail used. D) Culture flask used. E) Base medium used. F) Type of serum used G) Virus or gene engineering vehicle used. H) The overall use of cytokine cocktails during CAR-T manufacturing and I) the same information by country of trial registration. For individual parameters numbers may not add up to 296 for lack of reporting.

In 214 of 296 trials (72.3 %), we could confidently identify the intracellular costimulatory domains present in the CAR (Figure 5a). 120/214 (56.1 %) of these trials use 4-1BB and 58/214 (27.1 %) CD28. 19/214 (8.9 %) of the trials combined both (represented as CD28BB, also known as third generation CAR). In a few trials from 2016-2022, novel costimulatory signals or combinations are used, such as CD27 or OX40 (<1 %). Trials evaluating three or more costimulatory signals and/or directly comparing them against each other were designated as other.

An important determinant of CAR-T cell therapy quality is the source material. Out of the 296 sampled trials, 219 (74 %) informed on the starting material used for manufacturing (Figure 5b). 150/219 (68.5 %) of those used PBMCs gained via leukapheresis. The remaining studies (69/219, 30.5 %), enriched cells for specific subsets before further processing. The most common enrichments were done for CD3+ (29/219, 13.4 %) or a mixture of CD4/CD8+ cells (27/219, 12.5 %). Some studies also enriched exclusively CD8+ cells (4/219, <2 %) or highly purified, for instance CD25-/CD62L+ cells (6/219, <3 %).

Expansion and genetic modification of T cells requires prior activation^104^. This triggers cell division and is still indispensable for viral transduction. For example, transduction with VSV-G pseudotyped lentiviral vectors is reliant on the expression of its ligand, the LDL-R receptor, which is not constitutively expressed on naive T lymphocytes^105^. 189/296 of our subsampled trials reported on the activation method used (69.9 %, Figure 5c). 73/189 (38.6 %) trials used anti-CD3 antibody, either alone or in combination with feeder cells, IL-2 and or costimulatory signals. More recent trials make use of off-the-shelf clinical grade T cell activation reagents. 96/189 (50.8 %) trials use magnetic antibody-coated beads for T cell stimulation, typically Dynabeads. 13/189 (6.8 %) trials report on using Miltenyi’s colloidal polymeric nanomatrix conjugated to humanized CD3 and CD28 agonists (TransAct) as an alternative to antibodies or beads.

During CAR-T cells *ex vivo* manufacture, the type of culturing vessel may impact the quality of the final product. 31/296 trials (10.5 %) report on the cell culture vessel used (Figure 5d). 13/31 (41.93 %) of the trials use the CliniMACS closed cell culture platform, 11/31 (35.5 %) standard culture flasks or plates, 4/31 (12.9 %) gas permeable rapid cell expansion (G-rex) bioreactors and 3/31 (10 %) culture bags.

Finally, the culture medium supplies essential nutrients to T cells during *ex vivo* culture. Of 296 subsampled trials, 149 trials report on the cultivation media (Figure 5e). Media composition varies substantially. The most common media are X-VIVO-15 (58/149, 38.9 %) and AIM-V (32/149, 21.5 %) (Figure 5e). Other media include TexMACS (19/149, 12.8 %), RPMI1640 (17/149, 11.4 %), GT-T551 (6/149, 4 %), CTS Optimizer (4/149, 2.7 %), H3 (1/122), Immunocult-XF (1/122), KBM581 (1/122). Additionally, 77 of 296 trials report on the use of plasma or serum as culture media supplement. However, serum of bovine origin or other non-human sera comes with the potential risk of zoonotic pathogens^106^. One way to address this risk is the use of human serum (53/77, 68.8 %), or autologous plasma. 9/77 (11.7 %) trials used serum replacement or the use of serum-free media for cultivation (Figure 5f).

In order to express CAR on T cells, different gene delivery methods are employed. 233/296 trials disclosed their chosen strategy (Figure 5g). One trial reported to use transient gene expression methods. However, stable CAR expression may be important for long-term tumor control. Therefore, stable integration of the CAR into the genome via viral transduction of T cells remains the current method of choice. Retroviral transduction with retronectin as transduction facilitator was used from the early years of CAR-T cell trials (68/233, 29.2 %). With the development of 2nd and 3rd generation non-reproducing lentiviruses, this method has now become the most common gene delivery strategy in recent trials (142/233, 60.9 %). In spite of their proven utility, viral methods still account for a substantial share of costs, when it comes to manufacture GMP grade lentiviruses. Only 20/233, (8.6 %) of trials use alternative methods, including Piggy Bac and Sleeping Beauty transposases, which may be cheaper alternatives. These non-viral methods could thus help render therapy more accessible^36,107^. It is important to mention, that these strategies do not come without risks as there have been reports of patients developing CAR+ T cell lymphomas after Piggy Bac- generated CAR-T cell administration^108^. This has been attributed to PiggyBac’s preference for integrating into transcriptionally active regions, which increases the likelihood of insertional mutagenesis. In contrast, the Sleeping Beauty system exhibits a more random integration pattern, potentially reducing this risk.

Of 296 subsampled trials, 176 reported details on the specific cytokines used during the manufacturing process of CAR-T cells (59 %, Figure 5h). IL-2 was used in 114/176 (64.8 %) of production processes reporting on the utilized cytokines, followed by different dual and triple combinations of IL-7/IL-15 (25/176, 14.2 %), IL-2/IL-7/IL-15 (18/176, 10.2 %), IL-2/IL-15 (10/176, 5.7 %), IL-7/IL-15/IL-21 (2/176, 1.1 %) and others (<<3 %). China, the United States and Europe, where most trials are conducted, explore cytokines in diverse combinations (Figure 5i). A noteworthy finding is that only Seattle Children’s Hospital reports on use of IL-21 as part of their clinical manufacturing formulation within our dataset, which needs to be weighted when judging data on IL-21. Investigators from Australia and Japan rely on IL-2 and trials in the United Kingdom were run with IL-2 alone or in combination with IL-15. However, only two trials from Japan and four from Australia were included into this meta-analysis and thus do not allow conclusions towards geographical trends, except that cell therapy may still be in its early stages.

The analysis of 296 CAR-T cell therapy trials revealed key trends in manufacturing methods but a substantial proportion of studies failed to disclose key aspects of their protocol. Most trials (68.5%) used peripheral PBMCs from leukapheresis as the starting material, while some enriched specific subsets like CD3+ or CD4/CD8+ cells. Magnetic antibody-coated beads (50.8%) were the most common activation method, as well as next-generation cell culture systems are rapidly being implemented at a large scale. Stable CAR expression was predominantly achieved through lentiviral transduction (60.9%), with retroviral methods used less frequently (29.2%). For culture media, X-VIVO-15 (41.4%) and AIM-V (23%) were the most popular and the addition of IL-2 was highly favored.

In order to identify CAR-T manufacturing parameters, including cytokine composition, that could immediately inform potential patient outcome, we performed a correlative analysis among those parameters. We focused on analyzing the use of cytokines in correlation, namely with the activation method, base media, serum supplementation or gene delivery tool used during manufacturing. While generally the overlap of available accessory metadata is rather low, some correlations such as preferentially used media and cytokine cocktails, could still be observed.

To find factors most strongly associated with each other, we performed a correlation analysis (multiple Chi2 test) and displayed results in a matrix format (Supplementary Figure 1). Initially, some logical and historically influenced correlations had to be excluded. For example, generally, start date and status of trial correlate with the use of older methodologies. Also, the correlative index between disease type, authors and sponsors are the highest, indicating a strong dependency to investigators choices in the selection of the manufacturing process.

**Figure 6:**
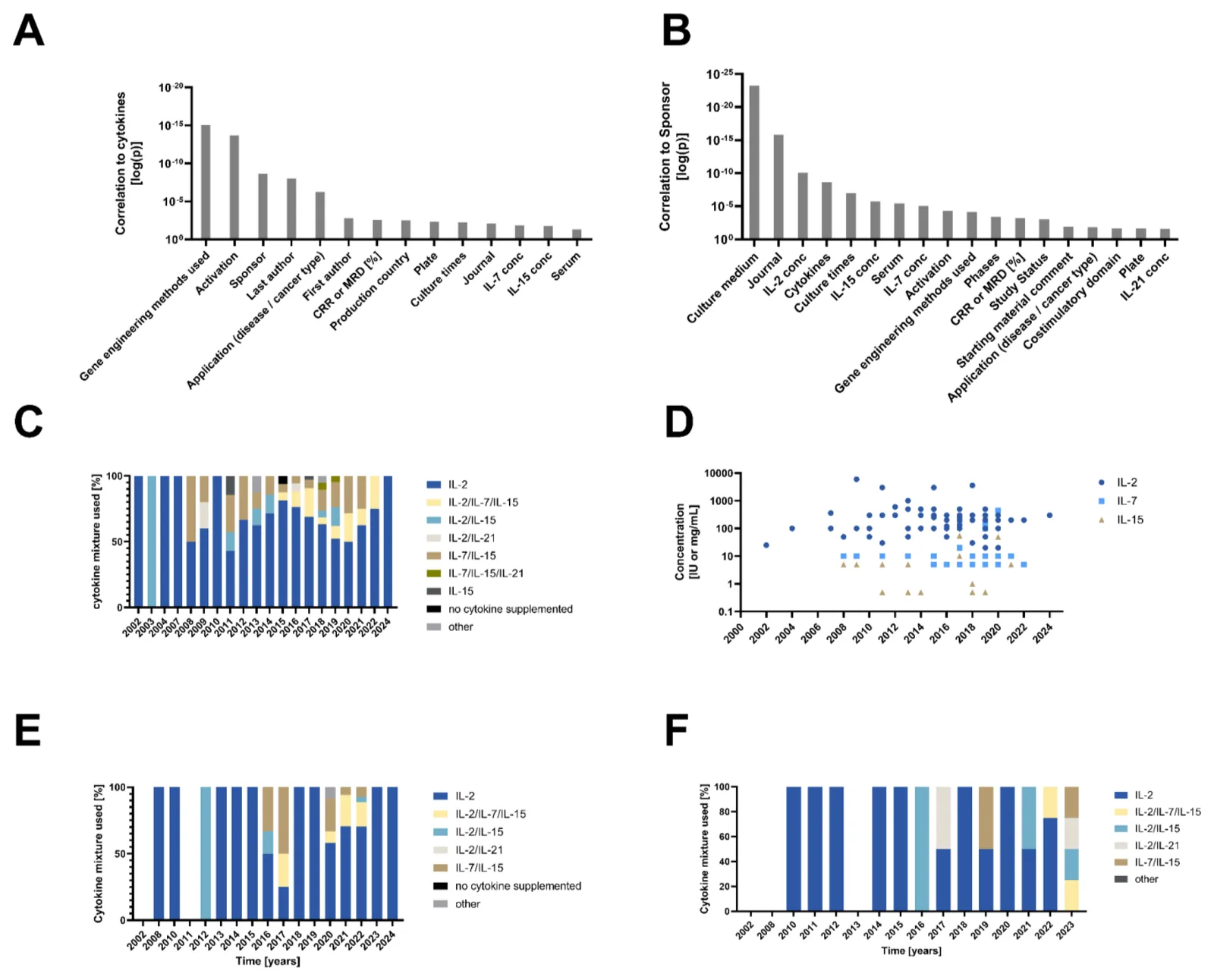
Correlative analysis of cytokines and other parameters assessed A) Correlation of the cytokines used with every other parameter ordered by significance (log p < 0.05). B) Correlation of the sponsors with every other parameter ordered by significance (log p < 0.05). C) The use of cytokines as a function of time highlighting the complexity of cytokine formulations used. D) The concentration of IL2 or IL7 or IL15, independent of formulation, as a function time. Each dot represents one study. E) Cytokine formulations used in hematological and F) in solid tumor clinical trials from 2002 to 2024.

Factors that specifically correlate most with the type of cytokine used for manufacturing are the gene engineering vehicle, activation method, sponsor and field of application (Figure 6a). This stems in part for the lack of availability of several methods and reagents for in-human use. Viral gene delivery systems including retro- and lentiviral vectors were strongly favored and only a few T cell activation cocktails were commercially available. It is also not surprising that inversely, several media composition parameters correlate most closely with the sponsor (Figure 6b). Particularly use of base medium, cytokines and their concentration as well as serum, activation methods and culture times are implemented differently in each laboratory with diverging- or perhaps moderately different results.

Early trials exclusively utilized IL-2. From 2008 onwards, some trials started to explore different combinations with other cytokines such as IL-7 and IL-15 (Figure 6c). Despite findings in 2011 that T cells, which are cultivated without IL-2 exhibit a favorable phenotype, IL-2 continues to be used in more than 50 % of trials to date.

An analysis of cytokine usage over time reveals an emerging trend in the utilization of more complex formulations including IL-7 and IL-15 and IL-21 in various combinations (Figure 6c). A discernible shift in the field indicates a convergence in IL-2 concentration compared to earlier trials (Figure 6d), with most trials using a median 200 IU/mL of IL-2. Outlier concentrations are heavily influenced by one laboratory and time-related reduction of IL-2 concentration coincides with publications highlighting the positive influences of IL-7 and IL-15^63^ as well as negative influences of IL-2 induced proliferation on CAR-T effector functions^51^. In 57% of cases, trials using high IL-2 concentrations (>1000 IU/mL) are targeting solid tumors, either against HER2 or CEA. When comparing hematologic malignancies versus solid tumors and observing the use of cytokines over time, it can only be concluded that cytokine solutions are becoming more diverse (Figure 6e,f). It can be speculated that while IL-2 still dominates hematological trials it may be disfavored towards solid tumor trials in the future.

**Figure 7:**
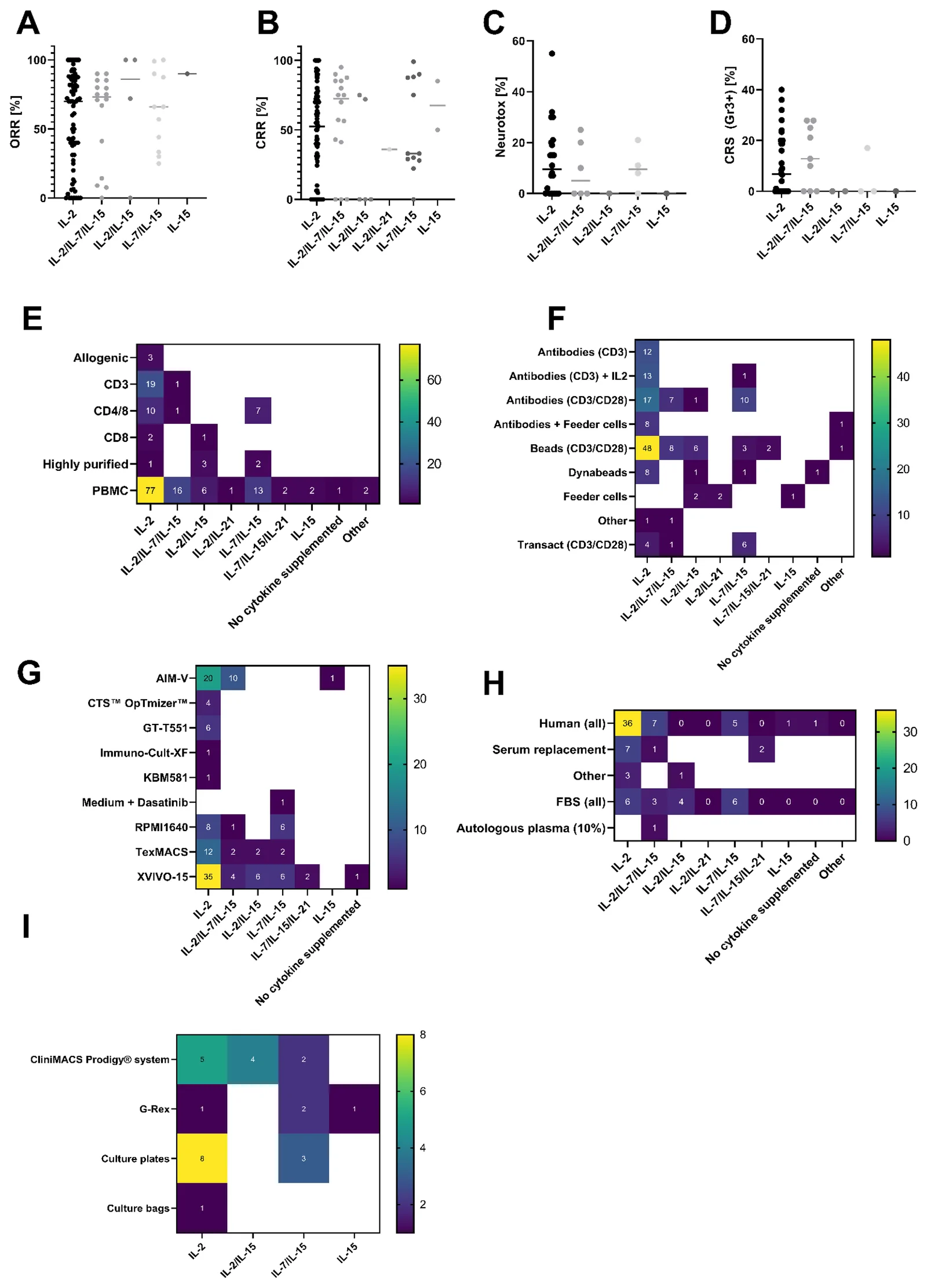
Correlative analysis of cytokines with other study components A) Distribution of cytokine use as a function of reported ORR and B) of CRR. C) Neurotoxicity and D) CRS by cytokines used. Data is shown as the percentage of the patient population. E) Heatmap showing most frequent combinations of cytokines and purification grade of starting material or F) activation method or G) base medium type or H) serum formulation or I) manufacturing platform.

Finally, we also correlate cytokines and response rates reported in clinical trials (Figure 7a,b). We are aware that comparisons between trials should be made with extreme caution. Typically, data can best be compared when trials are performed within a very similar patient population. We accept this as a limitation of this study and conclusions drawn from this dataset should be handled with caution and prospectively verified.

Trials with low overall response rate (ORR), regardless of cytokine use, belong to the ones with first generation CAR-T cells or those targeting solid tumors, particularly neuroblastoma, glioblastoma and melanoma. More complex cytokine cocktails were established later, thus ORR and complete response rate (CRR) associated with those trials may appear higher. Similarly, while toxicities tend to be associated with IL-2 usage, the dataset is not yet large enough to reach statistical significance in this regard (Figure 7c,d). Thus, given the comparably small data set with a large heterogeneity in decisive parameters, we cannot conclude on the use of one over the other cytokine towards patient outcome.

We further correlated cytokines across other important manufacturing parameters such as source- and purification state of lymphocytes, their activation method, the base-medium and serum usage. Simpler and older activation methods such as the exclusive use of anti-CD3 antibodies and use of feeder cells correlate strongly with IL-2 usage, presumably for historical reasons (Figure 7f). In contrast, newer technologies that include anti-CD28 antibodies or coated beads / nano matrices are more often paired with complex cytokine formulations.

For the same reasons, simpler cell culture vessels such as standard plates and flasks correlate with IL-2 use and/or single-cytokine use. Newer, more complex bioreactors and totally closed manufacturing systems are paired with complex cytokine formulations as well (Figure 7i).

A similar, inverse time relationship can be observed when looking at base media (Figure 7g,h). While older base-media such as RPMI, and X-Vivo and AIM-V are combined with almost any cytokine mixture, more recently commercialized base-media formulations that include serum-free solutions are quite traditionally combined with IL-2 exclusively. The only exception to this trend is TexMACS, which correlates to a mix of IL-2, IL-7 and IL-15 cytokine combinations.

**Figure 8:**
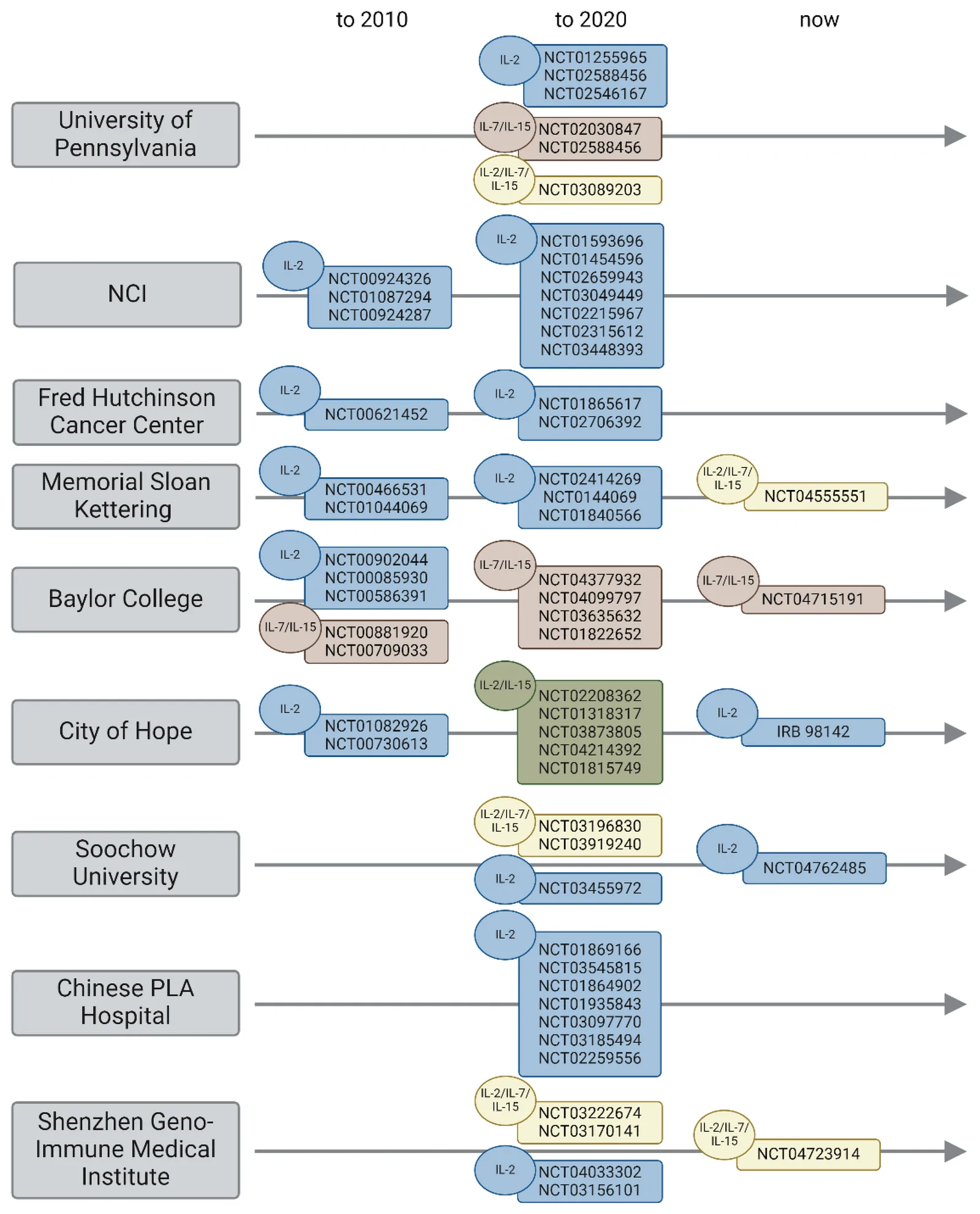
Key sponsors of CAR T clinical trials and cytokines they use over time Flow chart of most important sponsors, identified by the numbers of trials run in the subsampled data set ranked by the time periods between 2000-2010, 2011-2020 and 2020 until to date.

CAR-T cells originate from academic institutes long before big pharma sponsors jumped into the field. Since then, as clinical development of CAR-T cells is dominated by a small number of sponsors in China and the USA, we sought to evaluate trends in these sponsor groups specifically (Figure 8). Here, we made assumptions that investigators from one sponsor would likely collaborate or have a common cell manufacturing facility. It is not surprising that different researchers at the University of Pennsylvania have tested as well as reported on the use of several cytokine cocktails in the clinic. This is reflected in our dataset. In contrast, trials associated with National Cancer Institute (NCI) and Fred Hutchinson Cancer Center have not wavered from the use of IL-2 over time, as shown in our cohort. While data is scarce within the subsampled trials, it appears that investigators at Memorial Sloan Kettering and Baylor College of Medicine introduced or even fully switched to formulations including IL-7/IL-15. Chinese sponsors (Soochow University, PLA hospital, Shenzhen Medical Institute) appear to maintain IL-2-exclusive media formulations with some switching back to IL-2 from IL-2/IL-7/IL-15 formulations in recent years. This data highlights a lack of a consistent trend in the use of cytokines across sponsors. Though, admittedly this may change in the future as unpublished and undisclosed trials are advancing towards completion and reporting.

## Discussion

A downside of current clinical trial registration that impacts informative analyses, is that only a limited amount of information is disclosed to the public. The available information is insufficient to evaluate systematically therapeutic agents and allow cross-trial comparisons. Delays in public reporting led to massive gaps in our actual understanding of the studied therapies. For CAR-T cell therapy in particular, the quality of this “living drug” is dependent on a potentially infinite number of parameters that may be individually optimized. Centralized and non-standardized production leads to different protocols across centers, resulting in different qualities of the final product and thus potentially patient outcome, both of which are ultimately difficult to compare^101^. In an attempt to shed some lights on these aspects, we performed an analysis on meta-data extracted from 296 trial associated reports. Manufacturing details including starting material, gene engineering vehicle and cytokine use, but also response rates and toxicity scores were extracted from primary- and secondary sources and combined with basic parameters from clinicaltrials.gov. We are aware that conclusions drawn from comparisons across trials should be handled with care. Nevertheless, we believe that in an attempt to standardize processes and share learnings of pioneers in the field with the broader scientific community, this collection is warranted.

The investigator themselves emerges as major variable determining the parameters used across studies (Figure 6a,b), which similarly may bias the literature as some authors are overrepresented in our data set. As processes have become more sophisticated and multiple GMP-compliant products are launched by several suppliers, their use has diversified in later compared to earlier years. It is evident that current data prevents conclusions on clear advantages of any CAR-T manufacturing over the others. Going forward, there is no clear consensus on these processes and any differences remain nominal.

We outline that early on CAR-T manufacturing protocols heavily relied on IL-2 for its potent induction of antigen-independent T cell proliferation. After several preclinical scientific discoveries implicating IL-2 usage with unfavorable cell characteristics^64^, other common-y cytokines were screened to better CAR-T manufacturing. Although solid clinical evidence that factually supports negative influence of IL-2 is missing, IL-15 and IL-21 are generally seen as favorable and their usage is growing. In any case, clinical manufacturing use of IL-2 has not been discontinued, and the use of different cytokine cocktails continues to be controversially discussed. Unfortunately, side by side comparisons of different cytokine combinations are only found in preclinical studies and have been extensively studied in other meta-analyses^64,69,109^. An aspect to consider is the use of healthy donor T cells for preclinical validation of cytokine combinations. In the case of autologous CAR-T cell transfer, a starting material from a diseased patient dramatically differs from a healthy donor, potentially comprising cells of inferior quality.

Identification of optimal CAR-T manufacturing parameters, including cytokine composition and concentration based on the present data-set is challenging. For some categories, we could only assess a very limited number of trials and the overall diversity weakens or biases statistics. Policies on reporting of manufacturing are extremely heterogeneous, as in a business setting mostly considered a trade secret. A positive example is the academic team that developed ARI-0001, a CD19-CAR-T product approved for hospital-use in Spain^110^. They reported their manufacturing pipeline in detail, allowing more precise appreciation and eventually replication thereof. Yet another hurdle in attempting to align production parameters is that most manufacturers of approved products do not disclose their production processes and if they do so, the information is not tabularized or collected in a database. Thus, learnings from these very potent approved products cannot be applied generally in the field to improve patient outcome in unrelated CAR-T associated trials. Finally, clinical results are additionally biased by patient selection criteria, target and indication, which cannot be easily normalized and we mostly disregarded those in the report.

We believe that foremost, this report offers a representative overview of the field and trends in development. Regrettably, our efforts to establish correlations between various production factors and patient outcomes did not yield any relevant associations, likely because of missing information in a substantial amount of studies and – or the extreme diversity observed in our target parameters. As a result, we were unable to pinpoint any specific components that are likely to immediately contribute to improved patient outcomes. Nonetheless, this does not imply that the production process may be irrelevant to the final product quality, as evidenced by numerous previous studies^100,101^. At the same time, our summary is reassuring in the sense that the protocols used in the recent past do not seem to underperform compared to others, indicating that differences may be more subtle and that patients may not suffer from this landscape with limited harmonization. Although consensus has recently been challenged, as researchers at the University of Pennsylvania showed novel and groundbreaking evidence of IL-2 having a favorable effect compared to IL-7/IL-15 combinations on the long-term persistence and efficacy of CAR-T cells *in vivo*^68^.

In order to superimpose where the field of cell therapy development is heading, we also discuss key literature around next-generation CAR-T cell products. Particularly cytokine-armored CAR-T cell products and shortened or *in vivo* production processes hold great potential in drastically changing the existing cell therapy landscape for patient outcome^95,96,111–114^. While early trials using these armored CAR-T cells focused on maximizing cytotoxic activity, a more balanced approach with manageable safety features is favored^115,116^. By reducing waiting time for a cell therapy, imposed by *ex vivo* expansion, patients may be able to receive earlier treatment without bridging therapy. In conclusion, we want to foreshadow that in the distant future, CAR-T cell manufacturing may be entirely translocated into the patient’s own body. In fact, as previously mentioned, the first patient has already been treated with *in vivo* generated CAR-T cells^117^. At the same time, such development may call for even more work on the role of cytokines to identify precisely *in vivo* cytokine requirements for optimal activity in the absence of lymphodepletion. This potential development could most drastically reduce GMP-related production costs and improve patient outcomes.

For now, however, a more universal standardized production procedure has the potential to lower costs and improve patient outcome. This could positively influence a decentralized manufacturing model where manufacturing logistics and production are automated and streamlined, leading to faster end-to-end processes. However, to permit successful roll-out, a collection and establishment of multi-center manufacturing guidelines are needed. Another important requirement would be the detailed disclosure of protocols in future publications, which should be prioritized by journals. This would also allow for comparability of CAR-T cell development, which is currently almost impossible, as this field is biased by investigators preferences, rather than harmonization and firm evidence. Globally, it will be essential to enhance knowledge sharing between researchers and create a hub for scientific exchange. This will help streamline clinical development with appropriate standards. Sharing knowledge and letting scientists benefit from the learnings of pioneers in the field should be a given and we hope for transparency in sharing these results.

## Limitations of the study

There are several limitations implied with the complexity of this study: first, study quality is inherently related to the one of the underlying 296 trials; second, we are limited to published literature on PubMed that is correctly indexed as clinical trial related data. This may lead to an underrepresentation of trials, especially those published in smaller or non-indexed journals, conference abstracts, or other non-traditional formats. This study is also subject to publication bias and may favor positive data that may be more extensively reported. Phase I/II data is overrepresented. Because of the lag phase between study initiation and reporting of clinical data, there may be a lack of transparency regarding more recent manufacturing processes. Taking all these liabilities into consideration, conclusions drawn from our collection might falsely lead to scientific exploration in avenues that lack reporting. Finally, we acknowledge that comparisons of response rates across trials with different products, different disease indications and individual patient selection criteria, are extremely fragile, if not impossible. Thus, particularly correlations of any manufacturing factor with response rates and toxicity scores should not be seen as evidential and looked at with care. We hope that as the bulk of initiated studies will start reporting results publicly, the field may be endowed with a larger database for comparison and parameter analysis, and we pledge for a central collection or repository in the future.

## Supplementary Material

Supplementary Materials
