# Supplementary material for: The role of cytokines for clinical CAR-T cell manufacturing: systematic review and analysis of current evidence
Source: Nat Biomed Eng. Author manuscript; Available in PMC 2026 Jul 10. (PMC7619229; doi:10.1038/s41551-026-01703-w)
Supplement: Supplementary Materials [file EMS215942-supplement-Supplementary_Materials.pdf]

Supplementary Material

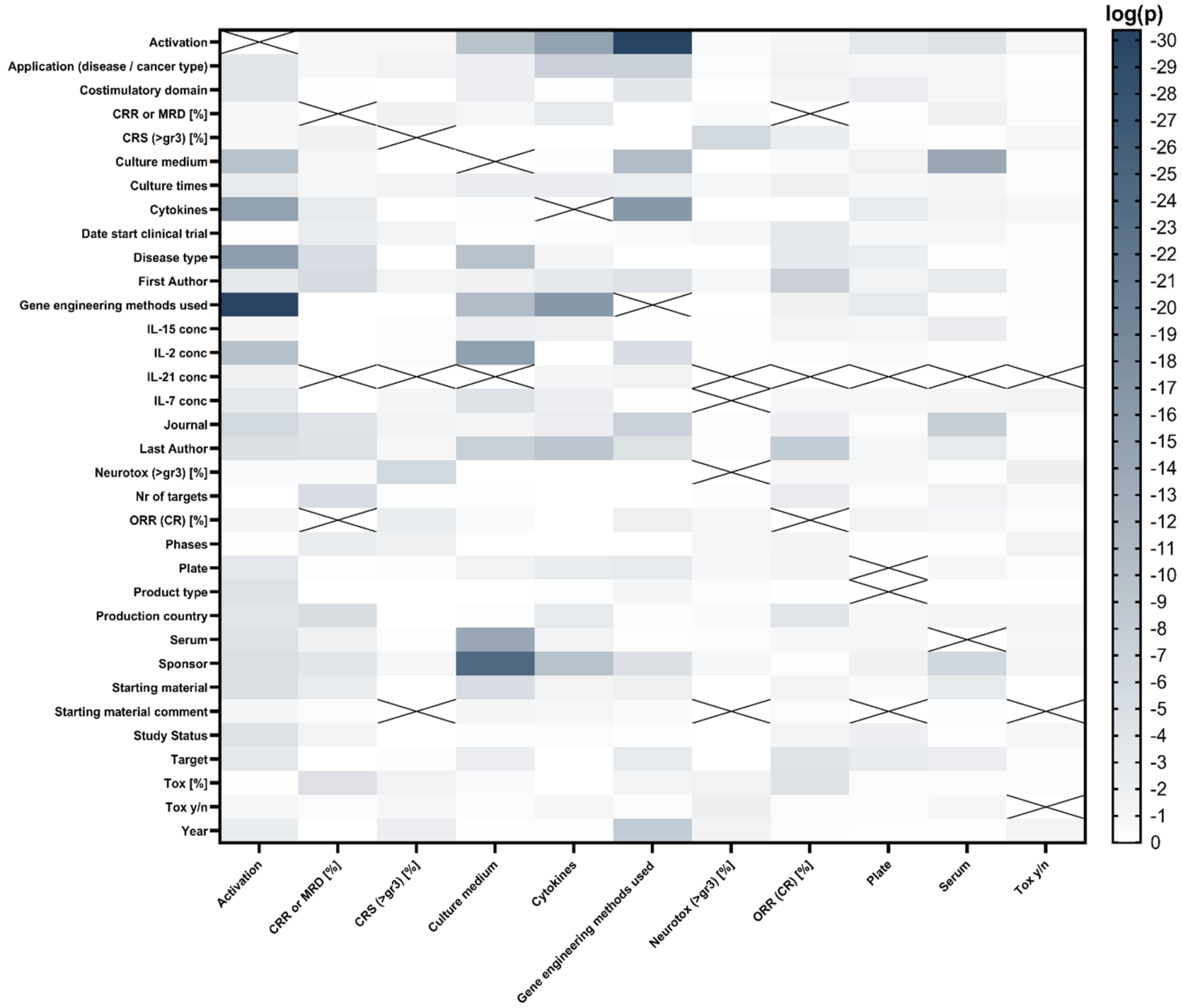

Supplementary Figure 1: Correlation matrix across the dataset
All parameters of the dataset were subjected to multiple Chi squared tests to evaluate possible relationships between the variables. The color indicates probability (log-p value) between two variables.
